# Fatal motorcycle crashes: a serious public health problem in Brazil

**DOI:** 10.1186/1749-7922-7-S1-S5

**Published:** 2012-08-22

**Authors:** Carlos Eduardo Carrasco, Mauricio Godinho, Marilisa Berti de Azevedo Barros, Sandro Rizoli, Gustavo Pereira Fraga

**Affiliations:** 1 Faculty of Medical Sciences, University of Campinas (FCM / UNICAMP) Campinas, SP, Brazil; 2 Division of Trauma Surgery, Department of Surgery, Faculty of Medical Sciences, University of Campinas (FCM/UNICAMP), Campinas, SP, Brazil; 3 Department of Public Health, Faculty of Medical Sciences, University of Campinas (FCM/UNICAMP), Campinas, SP, Brazil; 4 Departments of Surgery and Critical Care Medicine, Sunnybrook Health Sciences Centre, University of Toronto, Canada

## Abstract

**Introduction:**

The numbers of two-wheel vehicles are growing across the world. In comparison to other vehicles, motorcycles are cheaper and thus represent a significant part of the automobile market. Both the mobility and speed are attractive factors to those who want to use them for work or leisure. Crashes involving motorcyclists have become an important issue, especially fatal ones. Specific severe injuries are responsible for the deaths. Defining them is necessary in order to offer better prevention and a more suitable medical approach.

**Methods:**

All fatal motorcycle crashes between January 2001 and December 2009 in Campinas, Brazil, were analyzed in this study. Official data have been collected from police incident reports, hospitals’ registers and autopsies. Both incidents and casualties were analyzed according to relevant variables. The Injury Severity Score (ISS) was calculated, describing the most potentially fatal injuries.

**Results:**

There were 479 deaths; 90.8% were male; the mean age was 27.8 (range 0-73); 86.4% were conductors of the vehicles; blood alcohol was positive in 42.3%; 49.7% died at a hospital; 32.6% died at the scene; 26.1% of the accidents occurred at night, 69.1% were urban and 30.9% occurred on highways. The main causes of injury were collisions (63%) and falls (14%). The mean ISS was 38.5 (range 9-75). With regard to injuries, head trauma (67%) and thoracic trauma (40%) were the most common, followed by abdominal trauma (35%). Traumatic brain injury (67%) and hypovolemic shock (38%) were the most frequent causes of death.

**Conclusions:**

Alcohol was a significant factor in relation to the accidents. Head trauma was the most frequent and severe injury. Half of the victims died before receiving adequate medical attention, suggesting that prevention programs and laws should be implemented and applied in order to save future lives.

## Introduction

The number of motorcycles is increasing worldwide, particularly in developing countries. A World Health Organization (WHO) study on the Americas concluded that in countries like Brazil, Mexico, Canada and the United States [1], motorcycle crashes are responsible for 20-30% of all deaths due to trauma.

In Singapore, motorcycle crashes are responsible for 54% of all deaths caused by any motor vehicle accidents [2]. In Italy in 1997 [3], 20% of all deaths due to traffic accidents involved motorcycles while in the United States the number of deaths due to motorcycle crash increased 103% between 1997 and 2006 [4], numbering 2,300 deaths in 1994 and 4,000 in 2004 [5]. In the United Kingdom in 1998 [6] motorcycle crashes were responsible for 15% of all deaths or serious injuries by traffic accidents.

The number motorcycles has increased especially in large urban areas possibly due to increasing fuel costs, intense traffic and low purchase price for motorcycles [1,7-10]. Despite being considered dangerous, motorcycles are an attractive and cheap option for leisure and/or work, particularly in urban areas.

In Brazil, motorcycles are widely used to transport correspondence in high traffic urban areas by a special class of workers known as “*motoboys*”, as well as taxis (“*moto-taxis”*). Despite a few studies demonstrating the enormous impact in mortality of motorcycle crashes, this issue has been mostly neglected by scholars, the public and registries, and the extent deaths due to motorcycle accidents occur in Brazil remains unknown [11-13].

Despite the laws regulating the use of helmets, safety equipment and the practice of traffic safety most of these rules are blatantly ignored in Brazil by motorcycle drivers, which is unfortunately also observed in many other places in the world particularly developing countries [14]. It is essential to understand better the injuries, the causes leading to the accident and other important data in order to prevent and reduce all injuries, particularly the fatal ones.

The purpose of this study is to investigate the epidemiological aspects of the deaths in motorcycle crashes, to define the most frequent and severe injuries observed in these accidents and analyze the Injury Severity Score (ISS) [16] of the casualties. Secondary goals are to warn on the urgent actions in injury prevention and regulation required in order to reduce the number of deaths and serious injuries in the future.

## Material and methods

All motorcycle crashes within the borders of Campinas, in the period from 2001 to 2009, were included in this study. Data analyzed included whether the driver and/or passenger were involved, whether the victims died or survive and excluded occupants from other vehicles that might also been involved in the same crash. Accidents occurring on highways or within city streets were included.

Campinas has over 1 million inhabitants and is the 3^rd^ most populous city in the state of São Paulo and 14^th^ in Brazil. Over the last few years the population has grown by 1.2% per year while the motorcycles fleet grew by 4.9% per year [14]. Thus Campinas motorcycle fleet is growing 4 times faster than its population. In 2009, Campinas had 126% more motorcycles than in 2001 and 69% of the motorcycle crashes had at least one severely injured or dead victim [14]. Between 2000 and 2008, Marín-León *et al*. [15] observed that motorcycles in Campinas were responsible for the highest pedestrian fatality rate (4 deaths/1,000 accidents).

### Sources

After Institutional Review Board (IRB) approval, data were obtained through an official city institution in Campinas (*EMDEC – Empresa Municipal de Desenvolvimento de Campinas*) which controls and manages the traffic within the borders of the city. Casualties, injury severities and autopsy reports were individually analyzed at the Institute of Legal Medicine, whose records also contain medical reports and police bulletins.

### Collecting data

A specific form was developed to suitably collect all the information required: age, gender, place of accident, cause of accident, moments of accident and death, injury(ies), medical procedures carried out and blood alcohol (victims were considered intoxicated when the blood alcohol analyses were positive).

### Trauma indices

Both the Abbreviated Injury Scale (AIS) and Injury Severity Score (ISS) were calculated for all those included in this study.

### Statistical analyses

Continuous variables were expressed by their means. Categorical data were expressed as frequencies and percentages. Comparisons between groups were made using the Chi square test or the Fisher exact test for categorical variables as appropriate.

## Results

### Victims

Between 200 and 2009 479 people died as consequence of a motorcycle crash in the city of Campinas in Brazil. Most, 90.8% were male and 86.4% were the driver of the motorcycle. The mean age was 27.8 (range: 0-73); blood alcohol was positive in 42.24% of the victims (mean rate: 0.627 g/L), 49.7% died in a hospital, 32.6% at the scene and 17.7% on route to a hospital or the time of death was unknown.

### Accidents

**Figure 1:**
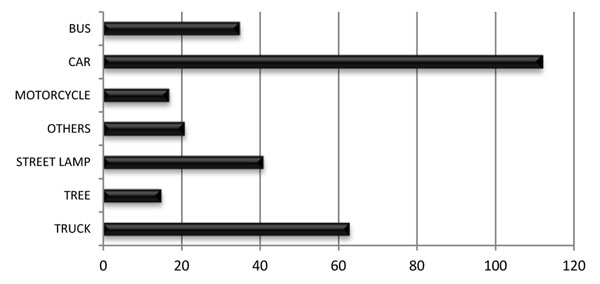
Distribution of collisions.

69.1% of the events occurred within the urban area and 30.9% on the highways. The most common accidents were collisions (63%) and falls (14%). The collisions involved cars in 37% of the occasions and trucks or buses in 32%. There were several different objects and vehicles that motorcycles collided with. Cars and large vehicles such as buses or trucks have emerged as the main protagonists (Figure 1). Street lamps, trees, walls, containers, animals and pedestrians were less common, but showed that even fixed objects can represent a serious danger to motorcyclists, especially when drivers are under the influence of alcohol. The most common time for accidents to occur was at night (between 6pm and midnight), when 26.1% of the collisions occurred.

### Injuries

**Figure 2:**
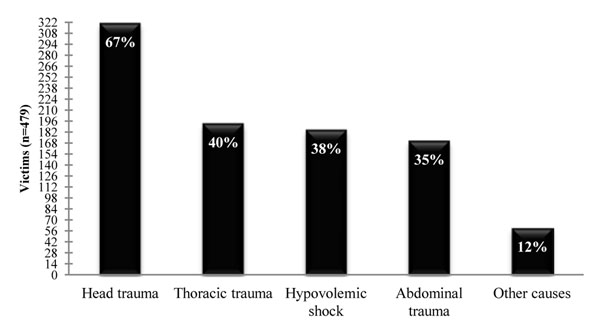
Major injuries found in fatal motorcycle victims.

Traumatic brain injury (TBI) was found as the most common injury (67%), followed by thoracic trauma and abdominal trauma (Figure 2). The results included injuries which occurred separately or together with other injuries. Hypovolemic shock was the cause of death in 38% of the cases, frequently associated with TBI.

### Trauma indices

**Figure 3:**
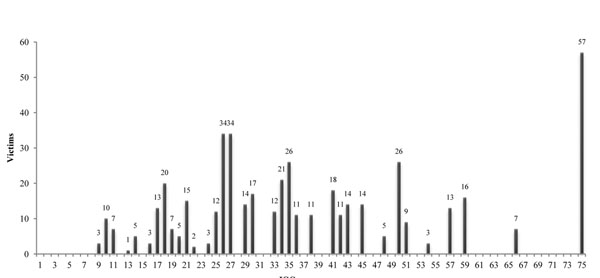
The trauma index ISS and its results.

Mean ISS was 38.51 (range: 9-75) and 11.89% of the victims had ISS = 75, the maximum value of the index (Figure 3). 80.4% scored ISS > 24 (very severe injuries).

**Figure 4:**
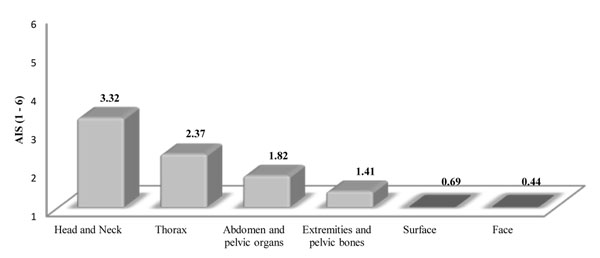
AIS and severity of injuries.

AIS shows that head and neck traumas are the most potentially fatal and severe injuries, followed by thorax, abdomen and pelvic organ injuries (Figure 4).

**Figure 5:**
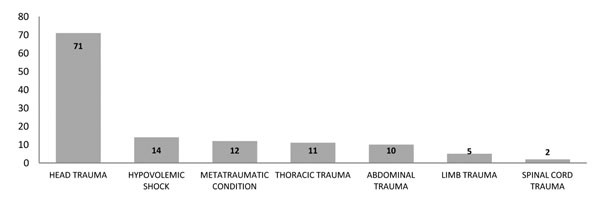
Causes of death of casualties with ISS 9-24.

ISS was higher for victims of highway crashes (median ISS: 41.0) than urban areas (Median ISS: 33.0) (p < 0.001). For the casualties who had ISS between 9 and 24 (n=94), the causes of death are illustrated in Figure 5.

### Time of death and its relations

**Figure 6:**
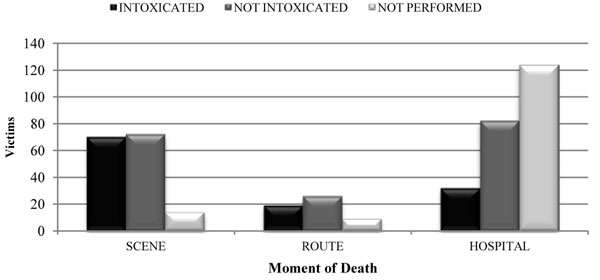
Relation of alcohol intoxication to moment of death.

1) Alcohol: most victims with positive blood alcohol died at the scene (p < 0.001); those with negative blood alcohol had similar time-of-death results when comparing the numbers of deaths at the scene or at a hospital (Figure 6).

2) ISS: Median ISS gradually decreases when considering the number of deaths at the scene (ISS=43), on route to a hospital (ISS=35) or at a hospital (ISS=30) respectively (p < 0.001).

### Surgical procedures

For those arriving alive at a hospital (238), 106 (44.53%) underwent surgery. Thoracic drainage was performed on 34 patients (32.1%), followed by a laparotomy on 29.2% and craniotomy on 23.6%. Orthopedic procedures, tracheotomies and other procedures were performed on just a few cases.

## Discussion

Most deaths observed in motorcycle crashes occur in young men and alcohol had a prominent role. Tests for blood alcohol levels are positive in many more motorcyclists than registered since these tests cannot be performed when there is either massive body destruction or urgent medical treatment. Literature has recognized that alcohol is the major contributing risk factor to fatal crashes [10,17]. Brazil has very strict laws on the question of driving under the influence of alcohol and this appears to be an influence in the reduction of accidents and deaths, as also demonstrated in other parts of the world [17].

Almost half of the patients reached a hospital alive, but the other half didn’t survive before pre-hospital teams had arrived at the scene of the accident, or before advanced trauma treatment could be put into practice. In accordance with local cultural habits regarding the consumption of alcohol, accidents frequently occur on Saturday nights.

Most accidents occurred in urban areas, but the most severe and potentially fatal injuries occurred on highways, where higher speeds are reached, which in turn exacerbates the severity of accidents.

When motorcycle accidents occur, injuries are often found in multiple body parts, being much more common than only in isolated ones. Even in relatively simple accidents, it is usual for wounds to the head and extremities to be found simultaneously. Associated with other injuries or not, head trauma was the most common injury found, despite the use of helmets being obligatory in Brazil, and this trend can be witnessed worldwide and is documented in associated literature [17-19]. This suggests that the trauma dynamics are so aggressive that even the use of appropriate equipment is not enough to avoid brain damage. Helmets, actually, change the forces applied on the head, but even so, those forces are extremely high, causing skin and muscle injuries when directly applied, or brain injuries when indirectly applied [18].

As the most frequent occurrence is blunt trauma, injuries to the intra-thoracic and intra-abdominal organs are common and cause serious bleeding, resulting in hypovolemic shock.

Trauma indices continue to be a very useful tool in evaluating trauma patients. In this study, for every ten victims, approximately eight suffered very severe injuries (ISS > 24), and fifty-seven casualties (11.9%) received maximum score (ISS = 75). This value is reached when potentially life-threatening injuries are found. Such results make clear that accidents involving motorcyclists usually result in serious damage to health or death. Something that must also be considered, however, is that almost 20% of the casualties had ISS < 24. In other words, those injuries considered minor or even moderate can result in death, depending on the causes of injury and the individuals’ health.

Regarding the six AIS body segments, motorcyclists receive the most severe injuries to the head and neck, followed by the thorax and abdomen. It’s notable that heart and liver injuries usually lead to severe or very severe stratification.

It may be further mentioned that ISS deviates according to the moment of death. As may be expected, deaths at the scene are likely to be more “severe” and deaths at a hospital not so. In general, ISS decreases as the victims near advanced trauma life support since it offers better diagnosis and treatment.

For those who reached hospital, survivability was improved via clinical support and/or surgical procedures. However, only 44.5% survived until surgery. According to injury frequency, surgical procedures were carried out on the thorax, abdomen and head. Other injuries, for example in extremities, are not usually life-threatening and were performed in some cases.

It is important to emphasize that 50% of the victims could not reach hospital, since they died instantaneously or en route to medical assistance. Helmets and other safety equipment sometimes have showed efficacy in reducing deaths or serious injuries, but solely, they are not sufficient to save lives [17,19]. When dealing with victims who suffer very severe and life-threatening injuries (80% of cases) and considering that half of those victims die before reaching hospital, it must be made clear that prevention is the most important action. Regarding this, laws regulating the use of helmets and the ingestion of alcohol are the most efficient prevention methods available and have had a notable impact on the numbers of accident and deaths. Another important point to note is that in areas in which there is no regular patrolling, even if mandatory laws exist, accidents have been increasing and hence the need for traffic control is urgent [20].

In Campinas, the number of deaths from traffic accidents has already exceeded that of homicides and other external causes of death, and motorcycles play a significant role in these statistics. Motorcycles are being used more and more all over the world and these concerns do not respect borders or private interests. Both developed and underdeveloped countries suffer the same results and therefore should work together, putting in practice appropriate actions to avoid those preventable deaths.

In conclusion, collisions involving motorcyclists frequently result in death. Young men are the most vulnerable group and there is a significant association with alcohol consumption, whose effects usually result in even more severe consequences. Most accidents take place in urban areas, but highways witness the most severe. Despite laws obligating the use of helmets and safety equipment, head trauma is the most frequent and severe injury for motorcyclists. Half of the victims die before reaching hospital, demonstrating the seriousness of the consequences of such accidents and not many victims, once in hospital, survive until surgery. Prevention programs and actions must be put in place, since solely a medical approach is insufficient to save many of these lives.

## Competing interests

The authors declare that they have no competing interests.
